# Prefrontal cortex oxygenation dynamics during dual-task walking in independently ambulatory patients with acute stroke: a comparison with healthy older adults

**DOI:** 10.3389/fnhum.2026.1945008

**Published:** 2026-09-22

**Authors:** Tomoya Takahashi, Kentaro Iwata, Natsuki Takahara, Erika Ohmori, Yukihiro Maekawa, Kanji Yamada, Sota Kobayashi, Nobuo Kohara, Atsuhiro Tsubaki

**Affiliations:** 1 Department of Rehabilitation, Kobe City Medical Center General Hospital, Kobe, Hyogo, Japan; 2 Graduate School of Health and Welfare, Niigata University of Health and Welfare, Niigata, Japan; 3 Department of Rehabilitation, Saiseikai Higashikanagawa Rehabilitation Hospital, Yokohama, Kanagawa, Japan; 4 Department of Physical Therapy, Faculty of Health Science, Juntendo University, Tokyo, Japan; 5 Institute for Human Movement and Medical Sciences, Niigata University of Health and Welfare, Niigata, Japan

**Keywords:** acute stroke, community ambulation, dual-task walking, near-infrared spectroscopy, prefrontal cortex, rehabilitation

## Abstract

**Background:**

Advances in stroke care have led to an increase in the number of patients who regain independent indoor ambulation; however, many still experience limitations when attempting to resume community activities. Altered prefrontal cortex (PFC) activity during dual-task walking (DTW) after stroke has been suggested as a contributing factor to difficulties with community ambulation. The purpose of this study was to compare PFC oxygenation dynamics and walking performance during DTW between independently ambulatory patients with acute stroke and healthy older adults.

**Methods:**

A total of 36 independently ambulatory patients with stroke within 14 days of onset (74.8 ± 6.3 years; 44% female) and 17 healthy older adults (71.4 ± 4.1 years; 41% female) were included in this cross-sectional observational study. Each participant performed normal walking as a single task (Walk-ST), serial subtraction as a single task (Serial-7-ST), and DTW tasks. We measured PFC oxygenation by assessing oxyhemoglobin (O_2_Hb) and deoxyhemoglobin (HHb) using a wearable near-infrared spectroscopy device and assessed walking speed, cadence, stride length, cognitive accuracy, and number of responses. Two-way repeated-measures analysis of variance was applied to the PFC oxygenation and performance outcomes, and the Hochberg method was used to adjust for multiple testing across related outcomes.

**Results:**

Significant task effects were observed for both O_2_Hb and HHb after Hochberg adjustment (both adjusted *p* < 0.01), whereas no significant group effects were detected. O_2_Hb was higher and HHb lower during Serial-7-ST and dual-task walking than during walking alone (all Bonferroni-adjusted *p* < 0.01), with no significant differences between Serial-7-ST and dual-task walking for either O_2_Hb (adjusted *p* = 0.20) or HHb (adjusted *p* = 0.15). Walking speed, cadence, and stride length showed significant group and task effects after Hochberg adjustment (all adjusted *p* < 0.05). No significant group differences were observed in cognitive accuracy or number of responses, although the number of responses showed a significant task effect (adjusted *p* = 0.04).

**Conclusion:**

Ambulatory patients with acute stroke exhibited poorer walking performance than did healthy older adults, despite showing no significant between-group differences in task-related PFC oxygenation dynamics or cognitive performance.

## 1 Introduction

Stroke remains a major cause of reduced healthy life expectancy (GBD 2021 Diseases and Injuries Collaborators, 2024). Over recent decades, stroke mortality and initial neurological severity have declined (Ananth et al., 2023; Toyoda et al., 2022), while advances in reperfusion therapy have improved functional outcomes in appropriately selected patients with cerebral infarction (Nogueira et al., 2018). Together, these trends have increased the clinical importance of supporting stroke survivors in resuming life in the community. In this context, regaining independence in indoor walking is an important rehabilitation milestone; however, it does not necessarily translate into successful community ambulation. In a previous study, although 81% of stroke survivors were independent indoor walkers at discharge from inpatient rehabilitation, only 27% met all physical criteria for unrestricted community ambulation (Blennerhassett et al., 2018).

Community ambulation requires more than the physical ability to walk; individuals must adapt to changing environments while allocating attention to concurrent cognitive demands, such as conversing, wayfinding, and monitoring traffic (Balasubramanian et al., 2014). Dual-task walking (DTW), defined as walking while concurrently performing a cognitive task, provides a controlled means of examining the ability to divide attention between walking and cognitive processing, thereby representing one component of community ambulation (Plummer and Eskes, 2015; Deblock-Bellamy et al., 2020). These demands are not fully captured by the ability to walk independently under predictable indoor conditions. In stroke survivors, cognitive-motor interference during DTW can impair walking and/or cognitive performance and may limit functional mobility and community participation (Plummer et al., 2013; Deblock-Bellamy et al., 2020). Therefore, evaluating DTW performance in addition to single-task indoor walking may provide clinically relevant information for establishing early rehabilitation goals aimed at safe community mobility.

The prefrontal cortex (PFC) plays a key role in DTW (Bishnoi et al., 2021). In a study of patients with chronic stroke (>6 months after stroke onset), DTW was associated with increased oxyhemoglobin (O_2_Hb) and decreased deoxyhemoglobin (HHb) relative to single-task walking, indicating increased PFC activation (Al-Yahya et al., 2016). Such activation may reflect greater executive demands and compensatory recruitment of PFC resources during walking after stroke (Al-Yahya et al., 2016). Because executive resources are limited, greater recruitment during DTW may be accompanied by poorer walking and cognitive performance when the combined task demands exceed the available capacity (Yogev-Seligmann et al., 2008). Patients with stroke may therefore rely more heavily on executive resources during DTW. According to this compensation framework, greater PFC recruitment in patients with stroke would be expected to accompany poorer walking and cognitive performance because fewer executive resources would remain available for task execution.

Near-infrared spectroscopy (NIRS) enables the assessment of cortical activity during overground walking without restricting natural gait patterns (Herold et al., 2018). However, most NIRS-based studies of DTW after stroke have focused on patients in the chronic phase. As more patients regain independent ambulation and return to the community soon after stroke onset, elucidating the characteristics of PFC activity and DTW performance in the acute phase has become increasingly important. During this phase, patients undergo rapid neurological and functional recovery, and the neural mechanisms underlying walking and cognitive performance may differ from those observed in the chronic phase. Therefore, findings from previous studies of chronic stroke may not be directly applicable to patients in the acute phase. Understanding these early changes may clarify the executive function demands required for a safe return to community life. Moreover, comparing these patients with healthy older adults living in the community enables researchers to distinguish stroke-specific patterns in PFC activation and performance from age-related changes.

This study aimed to compare PFC oxygenation dynamics and walking and cognitive performance during DTW between independently ambulatory patients with acute stroke and community-dwelling healthy older adults. The DTW condition combined walking with a cognitive task involving serial subtraction by sevens. We hypothesized that (1) independently ambulatory patients with acute stroke would exhibit greater PFC activation—reflected by increased O_2_Hb and decreased HHb—during DTW than healthy older adults and (2) these patients would demonstrate lower walking and cognitive performance during DTW than healthy older adults.

## 2 Methods

### 2.1 Participants

This study used a cross-sectional observational design and included two groups: patients with acute stroke and community-dwelling healthy older adults. Patients were consecutively included from the Stroke Care Unit of Kobe City Medical Center General Hospital in Hyogo, Japan, following admission for acute stroke. Acute stroke was defined as symptom onset within the previous 14 days and included three subtypes: cerebral infarction, intracerebral hemorrhage, and subarachnoid hemorrhage. Patients with stroke were eligible if they were aged ≥65 years, had experienced a first-ever stroke, had been independently ambulatory in the community before onset, and were able to walk independently on level indoor surfaces without assistance or supervision from another person, corresponding to a Functional Ambulation Category (FAC) score of ≥4 (Holden et al., 1984). Healthy older adults were recruited from a regional city in Japan and were eligible if they were aged ≥65 years, independently ambulatory in the community, and reported no health conditions meeting the prespecified exclusion criteria described below. The exclusion criteria were identical for both groups and included cognitive impairment (Mini-Mental State Examination [MMSE] score<21), other neurological disorders, uncontrolled hypertension or cardiovascular disease, lower-limb pain interfering with walking, severe obesity (body mass index [BMI] > 40), current corticosteroid use, respiratory disease requiring oxygen therapy, renal disease requiring dialysis, visual or vestibular impairment compromising safe ambulation, lower motor neuron disorders, fractures or joint replacement within the past 6 months, terminal cancer, inability to understand instructions in Japanese, and any condition the investigators deemed inappropriate for participation (Hawkins et al., 2018; Hermand et al., 2020; Chatterjee et al., 2019).

In accordance with the Strengthening the Reporting of Observational Studies in Epidemiology statement (von Elm et al., 2008), we defined the study design, setting, recruitment period, eligibility criteria, and data collection procedures. All participants provided written informed consent after receiving verbal and written explanations in accordance with the Declaration of Helsinki. The protocol was approved by the institutional ethics committee (approval number: zn230408).

The target sample size—36 patients and 18 healthy older adults—was determined in consultation with biostatisticians at the Kobe Biomedical Innovation Cluster. We reviewed the findings of Hawkins et al. (2018) when calculating the sample size; however, because estimates for our NIRS-based outcomes were limited, we determined the sample size based on feasibility and methodological requirements for counterbalancing task sequences rather than on a formal power calculation. Given the exploratory nature of this study, we adopted a pragmatic approach in consultation with biostatisticians. Additionally, counterbalancing the three tasks yielded six possible task orders. To enable balanced allocation across these orders using blocks of six, the target sample size in each group was set as a multiple of six. The healthy older adult group was included as a reference group for comparison. These considerations guided the final target sample size.

### 2.2 Measurements

The following demographic and clinical data were collected: age, sex, BMI, handedness, and use of walking aids. The following assessments were performed within 3 days before the NIRS measurement: 6-min walk distance (6MWD), maximal walking speed (10-m walk test), stair-climbing item of the Functional Independence Measure (FIM stair), FAC, MMSE, and Montreal Cognitive Assessment. For the four walking indicators, we additionally calculated the number and proportion of participants who met established community ambulation cutoffs (6MWD ≥ 367 m, maximal walking speed ≥0.80 m/s, FIM stair ≥6, and FAC ≥ 4) (Blennerhassett et al., 2018). For patients with stroke, the following additional clinical information was collected: stroke subtype, lesion location and side, National Institutes of Health Stroke Scale score on admission, days from onset to measurement, use of acute reperfusion therapy (for cerebral infarction only), modified Rankin Scale score at discharge, discharge destination, and details of the rehabilitation course.

### 2.3 Protocol

The trials were conducted on a rectangular walkway (16 m × 5 m). Each trial involved three personnel: the participant, researcher, and physical therapist positioned behind the participant to ensure safety. Based on previous studies (Yogev-Seligmann et al., 2008; Hawkins et al., 2018; Hermand et al., 2020; Chatterjee et al., 2019; Wang et al., 2023), three task conditions were used:

1. Normal walking performed as a single task (Walk-ST).
2. Serial-7 subtraction performed as a single task (Serial-7-ST).
3. DTW combining walking and serial-7 subtraction (DTW).

Serial-7 subtraction was selected to maintain methodological comparability with a previous NIRS study of dual-task walking in individuals with chronic stroke that used the same three task conditions (Chatterjee et al., 2019).

In the Walk-ST condition, participants were instructed to walk at a self-selected comfortable speed. In the Serial-7-ST condition, participants stood upright near a handrail and repeatedly subtracted seven from a random number between 91 and 99, verbalizing each response. When the subtraction reached 0, a new starting number between 91 and 99 was immediately provided. In DTW, participants performed Walk-ST and Serial-7-ST simultaneously, with instructions to allocate attention equally to both tasks. Each task was performed once for 70 s. This duration was selected to provide a 60-s active analysis period from 7 to 67 s after task onset, accounting for the approximate 6-s neurovascular delay, while ensuring that the analysis period ended before task termination. Tasks were separated by 60-s quiet-standing rest periods. The rest duration was based on the approximately 1-min quiet-standing baseline used before walking tasks in a comparable NIRS study of dual-task walking after stroke (Chatterjee et al., 2019) and was intended to allow the hemodynamic response to recover toward baseline before the subsequent task. Participants were instructed to maintain a consistent head height, avoid unnecessary conversation, and refrain from movements unrelated to task performance. To minimize order effects, participants within each group were assigned to the six possible task orders using computer-generated block randomization with a block size of six, with each task order assigned once within each block.

Before data acquisition, participants completed one familiarization lap of the walkway with all measurement equipment in place, including the NIRS probes, video camera, and audio recorder, and practiced Serial-7 subtraction starting from 100. The investigator confirmed that each participant understood the task procedures and provided additional explanations when necessary. After familiarization, participants were given sufficient time to rest before the recorded trials began. Participants began the tasks upon receiving the verbal cue “Start the task (Walk-ST, Serial-7-ST, or DTW)” and stopped immediately upon receiving the instruction to end the task. No countdown was provided to avoid inducing anticipatory PFC activation (Ishii et al., 2018).

### 2.4 Outcome measures

PFC oxygenation was measured using a wearable NIRS device (Hb133; ASTEM, Japan). Probes were placed over Fp1 and Fp2 according to the international 10–20 system. Source–detector distances were 30 mm and 35 mm, with wavelengths of 760 and 850 nm, enabling estimation of O_2_Hb and HHb (μmol/L) based on the modified Beer–Lambert law (Kocsis et al., 2006). The sampling frequency was 10 Hz. The device did not include short-separation channels, and concurrent systemic physiological signals, such as heart rate and respiration, were not recorded.

Task performance was recorded using a video camera mounted on the measurer’s head and an audio recorder attached near the participant’s collar. Video and audio recordings were analyzed offline by a single trained researcher who was not blinded to group membership or task condition. Walking and cognitive outcomes were calculated using the operational definitions described below. Walking outcomes (walking speed [m/s], cadence [steps/s], and stride length [m]) were obtained during Walk-ST and DTW. Walking speed was calculated as walking distance divided by time, cadence as the number of steps divided by time, and stride length as walking distance divided by the number of steps during the 7–67 s analysis period. Cognitive outcomes (accuracy [%] and number of responses [n]) were obtained during Serial-7-ST and DTW. Accuracy was calculated as the number of correct responses divided by the total number of responses, and the number of responses was defined as the total number of verbal responses produced during the same 7–67 s analysis period.

### 2.5 Data processing

During data acquisition, signal quality at Fp1 and Fp2 was assessed using the device’s sensor-error indicators and visual inspection of the real-time waveforms. If an error or poor-quality waveform was detected, the corresponding probe was repositioned. Participants were excluded if the problem persisted at either site or if data from either Fp1 or Fp2 in any of the three task conditions were missing or unusable because complete bilateral recordings across all three conditions were required for the repeated-measures analyses. No prespecified signal-to-noise ratio or cardiac-power threshold was applied. Raw NIRS signals were smoothed using an 11-point moving average, corresponding to an approximately 1.1-s window at the 10-Hz sampling rate. This low-pass finite impulse response filter was applied to attenuate high-frequency fluctuations relative to the slow task-related hemodynamic changes assessed over the 60-s analysis period (Pinti et al., 2019). The hemodynamic modality separation method was applied to mathematically decompose the measured O_2_Hb and HHb signals into estimated functional and systemic components based on the assumed negative relationship between O_2_Hb and HHb in the functional component and the positive relationship between these signals in the systemic component (Yamada et al., 2012). The estimated functional component was used in the subsequent analyses.

Baseline O_2_Hb and HHb values were defined as the mean values recorded during the final 10 s of standing rest before each task. For Serial-7-ST, both the baseline and task periods were performed while standing; therefore, calculating task-related O_2_Hb and HHb responses relative to the immediately preceding standing baseline minimized the tonic hemodynamic contribution associated with maintaining an upright posture. Task-related O_2_Hb and HHb values were calculated as the mean values from 7 to 67 s after task onset and expressed as changes from baseline. The analysis window began at 7 s to account for the approximately 6-s hemodynamic delay and extended for 60 s, ending 3 s before completion of the 70-s task (Hawkins et al., 2018; Chatterjee et al., 2019; Wang et al., 2023).

$$\Delta O_{2}\mathrm{Hb}\;(\text{ΔHHb})=\text{task}-\text{baseline}$$

Changes in O_2_Hb from ST to DTW were calculated as the difference between the O_2_Hb response during DTW and that during ST (Yogev-Seligmann et al., 2008).

$$\Delta O_{2}\mathrm{Hb}\;\text{during}\;\mathrm{DTW}=\Delta O_{2}\mathrm{Hb}\;(\mathrm{DTW})-\Delta O_{2}\mathrm{Hb}\;(\mathrm{ST})$$

Mean walking and cognitive performance values from 7 to 67 s were used to ensure consistency with the NIRS analysis intervals. Dual-task cost was calculated to quantify the relative change in performance between conditions (Chatterjee et al., 2019).

$$\text{Dual task cost}=\frac{\left(\mathrm{DTW}\;\text{performance}-\mathrm{ST}\;\text{performance}\right)}{\mathrm{ST}\;\text{performance}}$$

### 2.6 Statistical analysis

Continuous participation characteristics were assessed for normality with the Shapiro–Wilk test and compared between groups with independent *t*-tests or Mann–Whitney U tests, as appropriate. Categorical variables were expressed as *n* (%) and compared using *χ*^2^ tests. Before the primary analyses, side-related differences and bilateral associations across tasks were examined using two-way repeated-measures analysis of variance (ANOVA) [side (left, right) × task (Walk-ST, Serial-7-ST, DTW)] and Pearson correlation analyses (Hawkins et al., 2018; Chatterjee et al., 2019). For the preliminary ANOVAs, the Hochberg procedure was applied separately to the two hemodynamic outcomes for each ANOVA effect. For the Pearson correlation analyses, the Hochberg procedure was applied across the six correlation tests, and 95% confidence intervals (CIs) for Pearson’s r were calculated using Fisher’s z transformation. The distributions of ΔO_2_Hb and ΔHHb satisfied the assumption of normality according to the Shapiro–Wilk test. After these preliminary analyses, the mean of the left and right signals was used in all subsequent analyses.

The first hypothesis was tested using two-way repeated-measures ANOVA (group [acute stroke, healthy older adults] × task [Walk-ST, Serial-7-ST, DTW]) applied to ΔO_2_Hb and ΔHHb. The second hypothesis was tested using two-way repeated-measures ANOVA (group × task [ST, DTW]) applied to performance outcomes. Walking outcomes were compared between Walk-ST and DTW, whereas cognitive outcomes were compared between Serial-7-ST and DTW. Partial eta squared (partial η^2^) was reported as the effect size estimate for the ANOVA models, with two-sided 95% CIs calculated by inversion of the noncentral F distribution. To control the family-wise error rate across related outcomes, the Hochberg procedure was applied separately to the *p*-values for each ANOVA effect (group, task, and group × task interaction) within the PFC oxygenation, walking, and cognitive outcome families (Hochberg, 1988). When a Greenhouse–Geisser correction was required, the corrected *p*-value was entered into the Hochberg procedure. Bonferroni-adjusted pairwise comparisons were performed following significant omnibus task effects, and mean differences with Bonferroni-adjusted simultaneous 95% CIs were reported. For repeated-measures factors with more than two levels, sphericity was assessed using Mauchly’s test. When the assumption of sphericity was violated, Greenhouse–Geisser-corrected *p*-values were used.

The relationship between ΔO_2_Hb during DTW and dual-task cost was assessed using Spearman’s rank correlation because several performance-related variables did not satisfy the assumptions of normality. Spearman’s rank correlation coefficient (*ρ*) was reported as the effect size estimate, with bias-corrected and accelerated bootstrap 95% CIs calculated using 5,000 resamples. Because these analyses were exploratory and performed separately within each group, the Benjamini–Hochberg procedure was applied across the five correlation tests within each group to control the false discovery rate (Benjamini and Hochberg, 1995). The CIs for the ANOVA effect sizes and correlation coefficients were not adjusted for multiple comparisons unless otherwise specified.

All analyses were conducted using R software (version 4.1; R Core Team, 2021), and statistical significance was set at *p* = 0.05.

## 3 Results

### 3.1 Participant characteristics

Between May 2023 and January 2025, 86 patients with stroke and 48 healthy adults were screened. After excluding participants aged <65 years (45 patients, 5 volunteers) and those with missing NIRS data (5 patients and 26 volunteers), the final sample comprised 36 patients and 17 volunteers. Among the 26 healthy older adults excluded because of unsuccessful NIRS recordings, 18 were excluded because of sensor errors and eight because of noise contamination. Thus, the final analyzed sample of healthy older adults (*n* = 17) was one participant below the prespecified target sample size (*n* = 18). Table 1 summarizes the demographic characteristics and the walking and cognitive assessment results. The patient group was significantly older and demonstrated significantly lower 6MWD, maximal walking speed, FIM stair, and FAC values than the control group. Table 2 summarizes the clinical characteristics of the patients with stroke. Among healthy volunteers, demographic characteristics did not differ significantly between participants included in the final analysis (*n* = 17) and those excluded because of missing NIRS data (*n* = 26) (Supplementary Table S1).

**Table 1:** Baseline characteristics of participants.

Characteristic	Acute stroke (*n* = 36)	Healthy older adults (*n* = 17)	*p*-value
Age, years	74.8 ± 6.3	71.4 ± 4.1	0.047
Female sex	16 (44%)	7 (41%)	0.823
Body mass index, kg/m^2^	22.7 ± 3.5	23.3 ± 3.1	0.555
Right-handed	33 (92%)	15 (88%)	0.690
Functional status at study enrollment			
Walking aids			0.051
Without	29 (81%)	17 (100%)	
With	7 (19%)	0 (0%)	
6-min walking distance, m	384.1 ± 92.9	551.0 ± 72.3	<0.001
≥367	21 (58%)	17 (100%)	
<367	15 (42%)	0 (0%)	
Maximum walking speed, m/s	1.3 ± 0.3	1.9 ± 0.3	<0.001
≥0.8	34 (94%)	17 (100%)	
<0.8	2 (6%)	0 (0%)	
Functional independent measure-stair	6 (5, 7)	7 (7, 7)	<0.001
7	10 (28%)	15 (88%)	
6	15 (42%)	2 (12%)	
5	9 (25%)	0 (0%)	
4	2 (6%)	0 (0%)	
Functional ambulation categories	4 (4, 5)	5 (5, 5)	<0.001
5	17 (47%)	17 (100%)	
4	19 (53%)	0 (0%)	
Mini-mental state examination	28 (26, 30)	28 (27, 29)	0.931
Montreal cognitive assessment	25 (23, 25)	25 (22, 27)	0.401

Continuous variables are reported as mean ±SD or median (interquartile range), depending on their distribution, and categorical variables are reported as *n* (%). For statistical comparisons, continuous variables were analyzed using the unpaired t-test or Mann–Whitney U test based on the distribution, and categorical variables were analyzed using the chi-square test.

**Table 2:** Clinical characteristics of patients with acute stroke.

Characteristic	Acute stroke (*n* = 36)
Premorbid history	
Premorbid mRS	0 (0, 0)
Walking aids	
Without	35 (97%)
With	1 (3%)
Stroke history	
NIHSS score on admission	3 (1, 8)
Stroke type	
Cerebral infarction	29 (81%)
Intracerebral hemorrhage	6 (17%)
Subarachnoid hemorrhage	1 (3%)
Lesion location	
Cortical	2 (6%)
Subcortical	28 (78%)
Mixed	6 (17%)
Lesion side	
Right	14 (39%)
Left	16 (44%)
Bilateral	6 (17%)
Acute reperfusion therapy for cerebral infarction (*n* = 29)	13 (45%)
Discharge mRS	1 (1, 1)
Discharge destination	
Home	26 (72%)
Rehabilitation hospital	10 (28%)
Recovery and rehabilitation process, days	
Start of bedside rehabilitation	1 (1, 1)
Start of mobilization out of bed	1 (1, 2)
Start of walking training	2 (1, 2)
Independent walking	5 (3, 7)
Post-stroke onset on the measurement day, days	9.3 ± 3.5

Continuous variables are reported as mean ± SD or median (interquartile range), depending on their distribution, and categorical variables are presented as *n* (%). mRS, modified Rankin Scale; NIHSS, National Institutes of Health Stroke Scale.

### 3.2 Task-related changes in PFC oxygenation

Before averaging the left and right signals, side-related differences and bilateral associations were examined. After Hochberg adjustment across the two hemodynamic outcomes for each ANOVA effect, significant main effects of task remained for both ΔO_2_Hb and ΔHHb, whereas neither the main effects of side nor the side × task interactions were significant (all adjusted *p* > 0.05; Supplementary Table S2). Moreover, the left and right signals were strongly correlated across all task conditions for both ΔO_2_Hb and ΔHHb (all *r* ≥ 0.734, all adjusted *p* < 0.001; Supplementary Table S3). Based on these preliminary analyses, the mean of the left and right PFC signals was used in the subsequent analyses.

Figure 1A shows the time-course changes in O_2_Hb and HHb for each task. In both groups, O_2_Hb showed a slight initial increase followed by a gradual decline during Walk-ST, a continuous increase throughout Serial-7-ST, and rapid rise after task onset that subsequently plateaued during DTW. HHb exhibited trends opposite to those of O_2_Hb across all tasks in both groups. Figure 1B shows the statistical results of task-related changes in ΔO_2_Hb and ΔHHb, illustrating between-group comparisons and differences among Walk-ST, Serial-7-ST, and DTW. The statistical results are summarized in Table 3. For both ΔO_2_Hb and ΔHHb, the main effects of task were significant (both Hochberg-adjusted *p* < 0.01), whereas neither the main effects of group nor the group × task interactions were significant (all Hochberg-adjusted *p* ≥ 0.40). Pairwise comparisons showed that ΔO_2_Hb was significantly higher and ΔHHb was significantly lower during Serial-7-ST and DTW than during Walk-ST (all Bonferroni-adjusted *p* < 0.001). Neither outcome differed significantly between Serial-7-ST and DTW (both Bonferroni-adjusted *p* ≥ 0.15).

**Figure 1:**
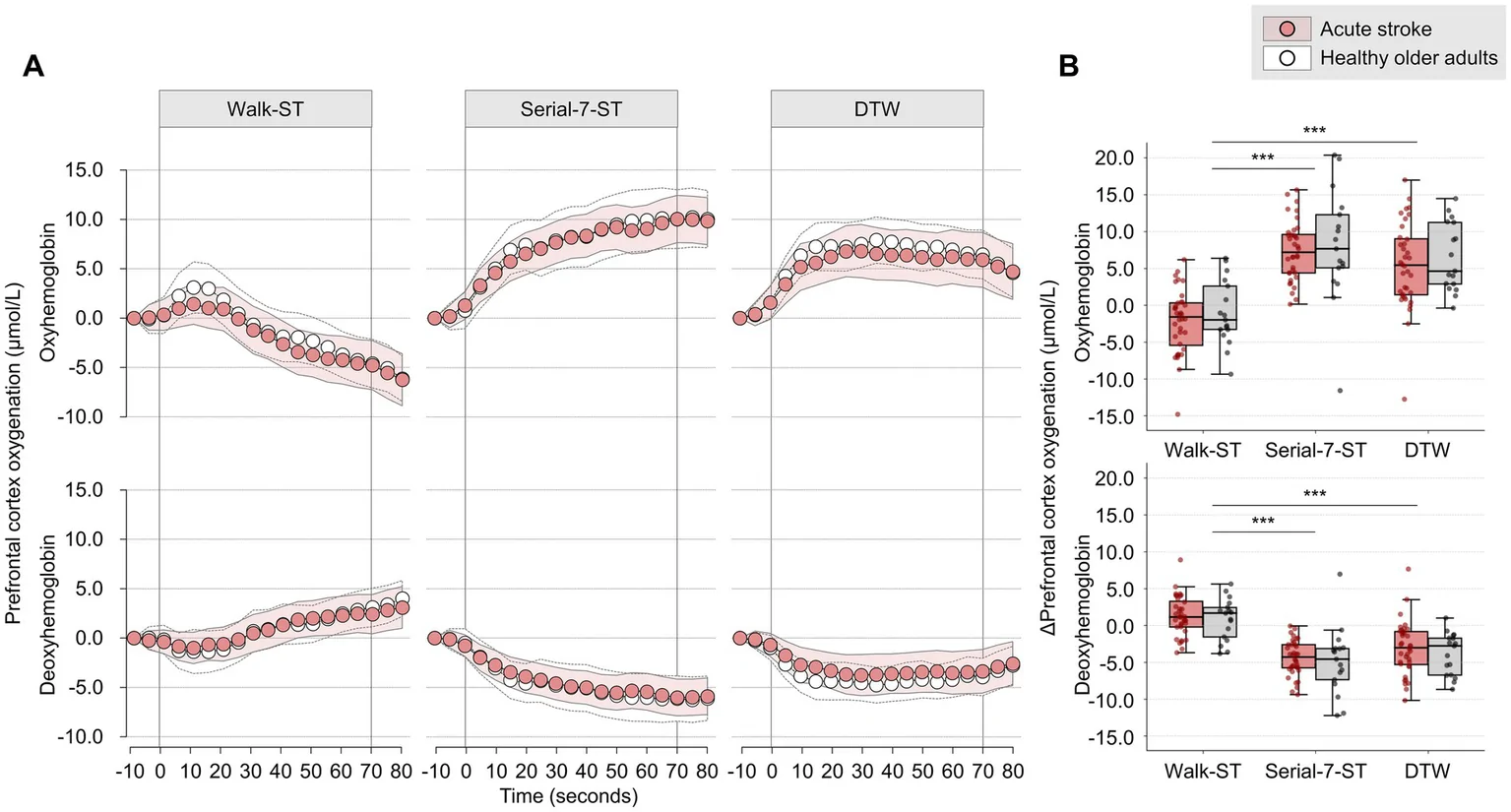
Task-Related Changes in Prefrontal Cortex Oxygenation in Acute Stroke vs. Healthy Older Adults. **(A)** Time-series changes in prefrontal cortex (PFC) oxygenation during normal walking performed as a single-task (Walk-ST), serial-7 subtraction performed as a single task (Serial-7-ST), and dual-task walking (DTW) in patients with acute stroke and healthy older adults. The upper panels show oxyhemoglobin responses, and the lower panels show deoxyhemoglobin responses. Shaded areas indicate the standard error of the mean. Red lines represent patients with acute stroke, and gray lines represent healthy older adults. **(B)** Task-related changes in oxyhemoglobin and deoxyhemoglobin across the three conditions. Box plots represent group distributions, with individual data points overlaid. Significant pairwise differences between task conditions based on Bonferroni-adjusted comparisons are indicated (***adjusted *p* < 0.001).

**Table 3:** Group and task effects on task-related changes in prefrontal cortex oxygenation.

Outcome	Effect	F (*df*)	Partial *η*^2^ (95% CI)	Adjusted *p*-value
A. Two-way mixed-design analyses of variance				
ΔO_2_Hb	Group	0.81 (1, 51)	0.02 (0.00 to 0.14)	0.40
	Task	57.03 (2, 102)	0.53 (0.40 to 0.63)	<0.01
	Group × task	0.04 (2, 102)	<0.01 (0.00 to 0.01)	0.96
ΔHHb	Group	0.72 (1, 51)	0.02 (0.00 to 0.13)	0.40
	Task	56.58 (2, 102)	0.53 (0.40 to 0.62)	<0.01
	Group × task	0.04 (2, 102)	<0.01 (0.00 to 0.01)	0.96

Outcome	Comparison	Mean difference (95% CI)	Adjusted *p*-value
B. Pairwise comparisons between tasks			
ΔO_2_Hb	Serial-7-ST − Walk-ST	9.32 (6.84 to 11.80)	<0.01
	DTW − Walk-ST	7.55 (5.50 to 9.60)	<0.01
	Serial-7-ST − DTW	1.77 (−0.57 to 4.11)	0.20
ΔHHb	Serial-7-ST − Walk-ST	−5.72 (−7.21 to −4.23)	<0.01
	DTW − Walk-ST	−4.53 (−5.79 to −3.27)	<0.01
	Serial-7-ST − DTW	−1.19 (−2.65 to 0.27)	0.15

Values are based on the mean of the left and right PFC signals. The Hochberg procedure was applied separately across the two hemodynamic outcomes (ΔO_2_Hb and ΔHHb) for each analysis-of-variance effect. The 95% CIs for partial *η*^2^ were calculated by inversion of the noncentral F distribution and were not adjusted for multiple comparisons. Pairwise comparisons were performed following a significant main effect of task; their p values and 95% CIs were adjusted using the Bonferroni method. Mean differences are expressed in μmol/L. CI, confidence interval; DTW, dual-task walking; O_2_Hb, oxyhemoglobin; HHb, deoxyhemoglobin; Serial-7-ST, serial-7 subtraction performed as a single task; Walk-ST, normal walking performed as a single task.

### 3.3 Task performance

Task performance results are shown in Figure 2, and the corresponding statistical results are summarized in Table 4. Walking speed, cadence, and stride length were significantly lower in the stroke group than in the healthy older adult group (all Hochberg-adjusted *p* = 0.04) and during DTW than during Walk-ST (all Hochberg-adjusted *p* < 0.01). No significant group × task interactions were observed for any walking outcome (all Hochberg-adjusted *p* = 0.55). Cognitive accuracy showed no significant main effects of group or task and no significant group × task interaction (all Hochberg-adjusted *p* ≥ 0.13). For the number of responses, a significant main effect of task was observed (Hochberg-adjusted *p* = 0.04), with fewer responses during DTW than during Serial-7-ST, whereas neither the main effect of group nor the group × task interaction was significant (Hochberg-adjusted *p* = 0.68 and 0.30, respectively).

**Figure 2:**
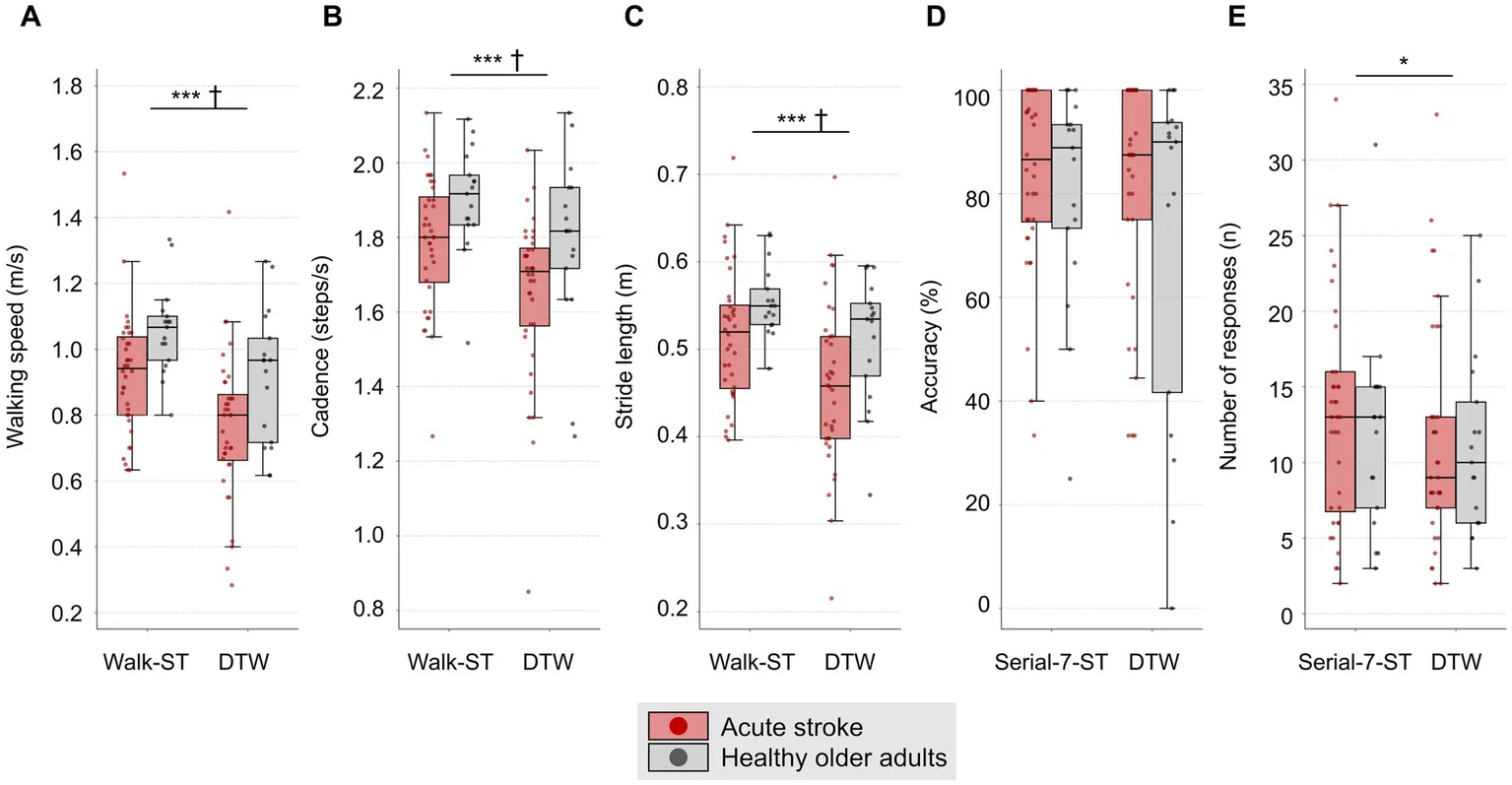
Task Performance in Acute Stroke vs. Healthy Older Adults. **(A–C)** Walking performance—walking speed **(A)**, cadence **(B)**, and stride length **(C)**—during normal walking performed as a single task (Walk-ST) and dual-task walking (DTW). **(D–E)** Cognitive performance—including accuracy **(D)** and number of responses **(E)**—during the serial-7 subtraction performed as a single task (Serial-7-ST) and DTW. Box plots represent group distributions with individual data points overlaid. Red plots indicate patients with acute stroke, and gray plots indicate healthy older adults. Significant task effects after Hochberg adjustment are indicated by *adjusted *p* < 0.05, **adjusted *p* < 0.01, ***adjusted *p* < 0.001, and significant group differences are indicated by ^†^adjusted *p* < 0.05.

**Table 4:** Group and task effects on walking and cognitive performance.

Outcome	Effect	*F* (1, 51)	Partial *η*^2^ (95% CI)	Adjusted *p* value
Walking speed	Group	6.61	0.12 (0.01 to 0.29)	0.04
	Task	84.17	0.62 (0.45 to 0.73)	<0.01
	Group × task	0.45	<0.01 (0.00 to 0.12)	0.55
Cadence	Group	5.11	0.09 (0.00 to 0.26)	0.04
	Task	40.40	0.44 (0.24 to 0.60)	<0.01
	Group × task	0.36	<0.01 (0.00 to 0.11)	0.55
Stride length	Group	4.47	0.08 (0.00 to 0.25)	0.04
	Task	45.76	0.47 (0.27 to 0.62)	<0.01
	Group × task	1.00	0.02 (0.00 to 0.15)	0.55
Cognitive accuracy	Group	1.39	0.03 (0.00 to 0.16)	0.49
	Task	2.43	0.05 (0.00 to 0.20)	0.13
	Group × task	1.09	0.02 (0.00 to 0.15)	0.30
Number of responses	Group	0.18	<0.01 (0.00 to 0.10)	0.68
	Task	5.73	0.10 (0.00 to 0.28)	0.04
	Group × task	1.13	0.02 (0.00 to 0.15)	0.30

Separate two-way mixed-design analyses of variance were conducted for the walking outcomes, with group (acute stroke vs. healthy older adults) as the between-subject factor and task (Walk-ST vs. DTW) as the within-subject factor, and for the cognitive outcomes, with group as the between-subject factor and task (Serial-7-ST vs. DTW) as the within-subject factor. The Hochberg procedure was applied separately across the three walking outcomes and the two cognitive outcomes for each ANOVA effect (group, task, and group × task interaction). Partial eta squared (partial *η*^2^) is reported as the effect-size estimate, with two-sided 95% CIs calculated by inversion of the noncentral F distribution. The 95% CIs were not adjusted for multiple comparisons. CI, confidence interval; DTW, dual-task walking; Serial-7-ST, serial-7 subtraction performed as a single task; Walk-ST, normal walking performed as a single task.

### 3.4 Relationships between ΔO_2_Hb during DTW and dual-task cost

Associations between ΔO_2_Hb during DTW and the dual-task costs for each performance measure are shown in Figure 3. In the acute stroke group, Spearman’s rank correlation coefficients were small for all performance measures (*ρ* = −0.21 to 0.05), and all 95% CIs included zero. None of these associations were statistically significant after Benjamini–Hochberg adjustment (all adjusted *p* ≥ 0.760). In the healthy older adult group, moderate correlations were observed for walking speed (*ρ* = 0.49, 95% CI [0.04 to 0.78]), cadence (*ρ* = 0.54, 95% CI [0.05 to 0.79]), and number of responses (*ρ* = −0.50, 95% CI [−0.82 to 0.05]). However, none of the five associations remained statistically significant after Benjamini–Hochberg adjustment (all adjusted *p* ≥ 0.073). Complete correlation coefficients, 95% CIs, and adjusted *p*-values are presented in Supplementary Table S4.

**Figure 3:**
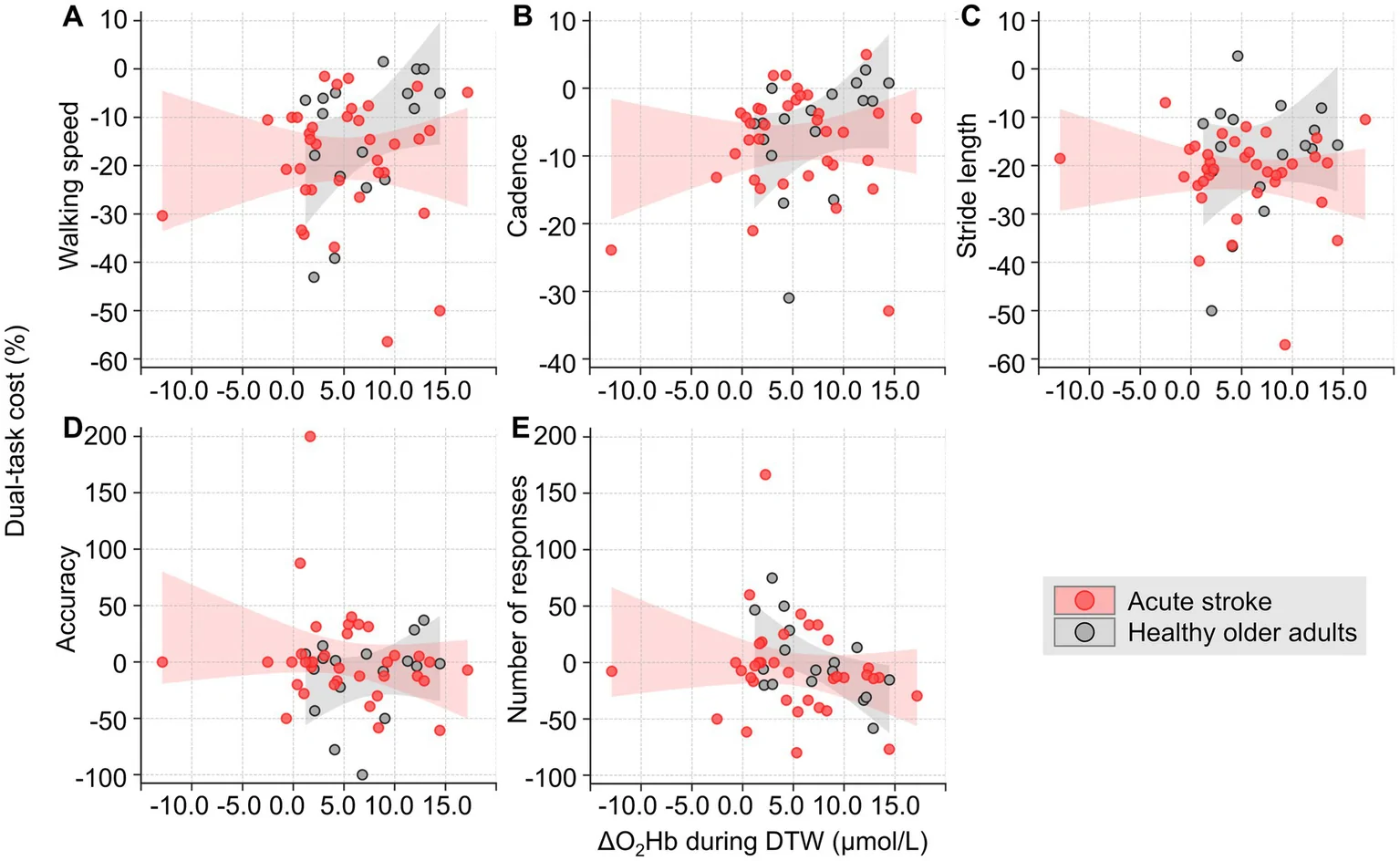
Associations between Prefrontal Cortex Oxygenation and Dual-Task Cost. **(A–C)** Associations between changes in prefrontal cortex (PFC) oxyhemoglobin during dual-task walking (ΔO_2_Hb during DTW) and dual-task costs in walking speed **(A)**, cadence **(B)**, and stride length **(C)** obtained from the walking tasks. **(D–E)** Associations between ΔO_2_Hb during DTW and dual-task costs in accuracy **(D)** and number of responses **(E)** obtained from the serial-7 subtraction tasks. Red filled circles represent patients with acute stroke, and gray-filled circles with black outlines represent healthy older adults. Shaded 95% confidence bands derived from least-squares regression models are displayed for descriptive purposes. Inferential analyses were based on Spearman’s rank correlations calculated separately for each group. None of the associations remained statistically significant after Benjamini–Hochberg adjustment across the five correlation tests within each group.

## 4 Discussion

We compared PFC oxygenation dynamics and task performance during DTW between patients with acute stroke and healthy older adults. Contrary to the first hypothesis, no significant group differences were observed in ΔO_2_Hb or ΔHHb after adjustment for multiple testing. However, both groups showed significantly higher ΔO_2_Hb and lower ΔHHb during DTW than during Walk-ST. Walking performance was significantly poorer in patients with stroke, whereas no significant group differences were observed in cognitive performance, partially supporting the second hypothesis. In the exploratory correlation analyses, several correlation coefficients were moderate in magnitude in the healthy older adult group; however, no associations were statistically significant in either group after correction for multiple testing. Hence, independently ambulatory patients with acute stroke demonstrated poorer walking performance than healthy older adults, despite no detectable between-group differences in task-related PFC oxygenation responses during DTW.

### 4.1 PFC oxygenation during DTW in acute stroke

These findings suggest that independently ambulatory patients with acute stroke may experience increased PFC activation during DTW to a level comparable to that of healthy older adults. No significant group differences in ΔO_2_Hb or ΔHHb were observed across tasks, and both groups showed increased ΔO_2_Hb and decreased ΔHHb during DTW relative to Walk-ST. These PFC oxygenation dynamics suggest that patients with acute stroke who are capable of independent indoor walking may retain sufficient executive resources during normal walking to support increased PFC activation during DTW. However, methodological factors may also have limited the sensitivity to detect a true group difference; therefore, the null finding should be interpreted cautiously.

The degree of gait impairment after stroke may influence PFC activation during DTW. In a study in which 55% of the patients required supervision for safe ambulation (FAC = 3), PFC O_2_Hb increased during single-task walking but did not increase further when a cognitive task was added during DTW (Hermand et al., 2019). Conversely, in another study in which 82% of the patients walked independently (FAC ≥ 4), DTW induced significant increments in O_2_Hb (Nosaka et al., 2023). In the present study, all patients were independently ambulatory on level ground (FAC ≥ 4), which was more comparable to the walking group examined by Nosaka et al. (2023). These findings suggest that walking ability may contribute to variability in PFC responses to additional cognitive demands during walking.

### 4.2 Task performance and PFC oxygenation–performance relationships during DTW

Patients with acute stroke demonstrated poorer walking performance than healthy older adults during Walk-ST and DTW, indicating that independent indoor ambulation did not necessarily correspond to performance levels comparable to those of healthy older adults. Moreover, correlations between DTW-related changes in ΔO_2_Hb and task performance were observed only in healthy older adults. Among healthy older adults, greater increments in ΔO_2_Hb were associated with smaller dual-task-related reductions in walking speed and cadence and with greater reductions in the number of responses. However, none of these associations remained statistically significant after adjustment for multiple testing. Therefore, these correlations should be interpreted cautiously as exploratory patterns and do not provide sufficient evidence that the relationship between task-related PFC oxygenation responses and dual-task performance differs between patients with acute stroke and healthy older adults.

Several studies involving patients with subacute or chronic stroke have reported significant relationships between PFC O_2_Hb responses and walking and cognitive performance during DTW (Mori et al., 2018; Chatterjee et al., 2019; Nosaka et al., 2023), reflecting the role of executive functions in supporting effective performance regulation (Yogev-Seligmann et al., 2008). In contrast, none of the associations examined in the present study remained statistically significant after correction for multiple testing. Furthermore, in community-dwelling older adults, PFC O_2_Hb decreased across three repeated DTW trials within a single session, while walking and cognitive performance improved, suggesting within-session learning and improved PFC efficiency (Holtzer et al., 2019). Patients who received rehabilitation and accumulated daily life experiences after stroke may have more refined performance-regulation mechanisms during DTW than those in the acute phase. Additionally, in the patients included in this study, automatized gait mechanisms, including brainstem locomotor regions and spinal central pattern generators (Clark, 2015), may have enabled walking with relatively low executive demands during DTW.

Collectively, these findings suggest that the relationship between cortical oxygenation responses and task performance may vary according to the stage of stroke recovery and walking ability, although this possibility could not be confirmed by the present exploratory analyses. Together with previous evidence that repeated DTW trials may improve the efficiency of PFC-mediated executive recruitment (Holtzer et al., 2019), the present results highlight the need for longitudinal and interventional studies to determine whether rehabilitation aimed at promoting more efficient executive recruitment during walking can improve DTW performance in independently ambulatory patients in the acute phase of stroke.

### 4.3 Study limitations

This study has several limitations. First, NIRS measurements were limited to Fp1 and Fp2, precluding localization of the observed responses to specific PFC subregions (Lim et al., 2022). Although the hemodynamic modality separation method was applied to reduce systemic physiological fluctuations, residual extracerebral and systemic influences during overground walking and verbal responding could not be completely excluded because short-separation channels and direct monitoring of superficial blood flow or systemic physiology were unavailable (Yamada et al., 2012; Yücel et al., 2021). Accordingly, the observed O_2_Hb and HHb changes should be interpreted as PFC oxygenation dynamics rather than direct evidence of spatially localized cortical activation. Furthermore, each condition was assessed in a single 70-s trial, without evaluation of test–retest reliability or within-condition averaging, which may have reduced sensitivity to group and group × task effects; future studies should use repeated shorter blocks (Binder et al., 2026). Second, standing balance and postural sway were not independently assessed; therefore, balance-related contributions to PFC oxygenation during Serial-7-ST cannot be excluded, despite using the immediately preceding standing period as the baseline. Clinical assessments performed within 3 days before NIRS acquisition may also not have fully reflected patients’ status on the testing day, although they were used only for sample characterization. Third, despite randomized task order and the absence of a countdown, the fixed 60-s inter-task interval may not have eliminated anticipatory responses or physiological fluctuations associated with the task schedule. Fourth, no *a priori* power calculation was performed, and the sample size was determined based on counterbalancing requirements and practical feasibility. The final healthy older adult sample (*n* = 17) was also one participant below the prespecified target (*n* = 18). Thus, small group differences may not have been detected, and the nonsignificant group effects should be interpreted cautiously. Fifth, many healthy older adults were excluded because of missing or unusable NIRS data. Although measured baseline differences between included and excluded participants were negligible to small (Supplementary Table S1), the high exclusion rate may have reduced control-group representativeness and statistical power and introduced selection bias. Sixth, age was not included as a covariate despite the significant group difference; therefore, residual confounding by age cannot be excluded. Finally, the cross-sectional design precludes causal interpretation of the associations between PFC oxygenation and task performance, and the exploratory correlation findings require confirmation in larger longitudinal studies. Nevertheless, these findings provide a basis for understanding the relationship between task-related PFC oxygenation responses and task performance during dual-task walking in ambulatory patients with acute stroke and may help inform future rehabilitation approaches aimed at improving community mobility after stroke.

## 5 Conclusion

Independently ambulatory patients with acute stroke showed no significant between-group differences in task-related PFC oxygenation dynamics during dual-task walking despite poorer walking performance than healthy older adults. These findings provide new insight into the relationship between cortical oxygenation dynamics and task performance during dual-task walking in the acute phase of stroke.

## Data Availability

The raw data supporting the conclusions of this article will be made available by the authors upon reasonable request.
