# Supplementary material for: Prefrontal cortex oxygenation dynamics during dual-task walking in independently ambulatory patients with acute stroke: a comparison with healthy older adults
Source: Front Hum Neurosci. 2026 Sep 22;20:1945008. doi: 10.3389/fnhum.2026.1945008 (PMC13640302; doi:10.3389/fnhum.2026.1945008)
Supplement: Supplementary file 1 [file Table_1.DOCX]

Supplementary Material

# 1 Supplementary Table S1. Baseline Characteristics of Healthy Older Adults Included in the Final Analysis and those Excluded Because of Missing NIRS Data

| Variable | Included  (n=17) | Excluded  (n=26) | Effect size  (95% CI) | *p*-value |
| --- | --- | --- | --- | --- |
| Age, years | 71.4±4.1 | 72.0±3.5 | -0.144  (-0.744 to 0.458)^a^ | 0.641 |
| Female sex | 7 (41%) | 11 (42%) | 0.011  (0.000 to 0.199)^b^ | 0.941 |
| Body mass index, kg/m^2^ | 23.3±3.1 | 23.4±3.9 | -0.020  (-0.620 to 0.580)^a^ | 0.948 |
| Right-handed | 15 (88%) | 25 (96%) | 0.152  (0.000 to 0.451)^b^ | 0.319 |
| Functional status at study enrollment |  |  |  |  |
| Walking aids |  |  | － | － |
| Without | 17 (100%) | 26 (100%) |  |  |
| With | 0 (0%) | 0 (0%) |  |  |
| 6-min walking distance, m | 551.0±72.3 | 549.0±81.5 | 0.024  (-0.576 to 0.624)^a^ | 0.938 |
| ≥367 | 17 (100%) | 26 (100%) |  |  |
| <367 | 0 (0%) | 0 (0%) |  |  |
| Maximum walking speed, m/s | 1.9±0.3 | 1.8±0.3 | 0.097  (-0.504 to 0.697)^a^ | 0.753 |
| ≥0.8 | 17 (100%) | 26 (100%) |  |  |
| <0.8 | 0 (0%) | 0 (0%) |  |  |
| Functional independent measure-stair | 7 (7, 7) | 7 (7, 7) | 0.113  (-0.120 to 0.308)^c^ | 0.367 |
| 7 | 15 (88%) | 20 (77%) |  |  |
| 6 | 2 (12%) | 6 (23%) |  |  |
| 5 | 0 (0%) | 0 (0%) |  |  |
| 4 | 0 (0%) | 0 (0%) |  |  |
| Functional ambulation categories | 5 (5, 5) | 5 (5, 5) | － | － |
| 5 | 17 (100%) | 26 (100%) |  |  |
| 4 | 0 (0%) | 0 (0%) |  |  |
| Mini-mental state examination | 28 (27, 29) | 28 (25, 29) | 0.111  (-0.249 to 0.430)^c^ | 0.545 |
| Montreal cognitive assessment | 25 (22, 27) | 25 (21, 27) | 0.066  (-0.295 to 0.412)^c^ | 0.726 |

Data are presented as mean ± standard deviation, median (25th, 75th percentiles), or n (%), as appropriate. Between-group comparisons were performed using the unpaired t-test, Mann–Whitney U test, or Pearson’s chi-square test, as appropriate. Effect sizes are presented with 95% confidence intervals (CIs): ^a^ Hedges’ g for unpaired t-tests; ^b^ Cramér’s V for Pearson’s chi-square tests; and ^c^ rank-biserial correlation for Mann–Whitney U tests. Positive values of Hedges’ g and rank-biserial correlation indicate higher values or ranks in the included group, whereas Cramér’s V is non-directional. The 95% CIs were calculated using the noncentral t distribution for Hedges’ g, the noncentral chi-square distribution for Cramér’s V, and a bias-corrected and accelerated bootstrap with 10,000 resamples for rank-biserial correlation. Effect sizes were not estimable for variables without between-participant variation and are indicated by an em dash (—). Of the 26 excluded participants, 18 were excluded because of sensor errors and eight because of noise contamination.

# 2 Supplementary Table S2. Results of Two-Way Repeated-Measures ANOVA for Left–Right Consistency of PFC Signals

| Variable | Effect | F | df | partial η^2^ | 95% CI for partial η^2^ | Unadjusted *p*-value | Adjusted *p*-value |
| --- | --- | --- | --- | --- | --- | --- | --- |
| ΔO_2_Hb | Side | 0.002 | 1, 52 | <0.001 | 0.000 to 0.013 | 0.965 | 0.965 |
|  | Task | 71.307 | 2, 104 | 0.578 | 0.456 to 0.667 | <0.001 | <0.001 |
|  | Side × task | 2.666 | 2, 104 | 0.049 | 0.000 to 0.141 | 0.074 | 0.074 |
| ΔHHb | Side | 0.002 | 1, 52 | <0.001 | 0.000 to 0.013 | 0.965 | 0.965 |
|  | Task | 68.040 | 2, 104 | 0.567 | 0.442 to 0.658 | <0.001 | <0.001 |
|  | Side × task | 3.301 | 2, 104 | 0.060 | 0.000 to 0.157 | 0.041 | 0.074 |

Results of the two-way repeated-measures analysis of variance examining the effects of side (left vs. right prefrontal cortex) and task (normal walking performed as a single task [Walk-ST], serial-7 subtraction performed as a single task [Serial-7-ST], and dual-task walking [DTW]) on task-related changes in oxyhemoglobin (ΔO_2_Hb) and deoxyhemoglobin (ΔHHb). Partial eta squared (partial η^2^) is reported as the effect-size estimate, with two-sided 95% confidence intervals (CIs) calculated by inversion of the noncentral F distribution. The Hochberg procedure was applied separately across the two hemodynamic outcomes (ΔO_2_Hb and ΔHHb) for each ANOVA effect (side, task, and side × task interaction). Both unadjusted and Hochberg-adjusted p-values are presented. Statistical inference was based on the adjusted p-values. The 95% CIs were not adjusted for multiple comparisons.

# 3 Supplementary Table S3. Correlation between Left-Right PFC Signals Across Tasks

| Variable | Task | r | 95% CI for r | df | Unadjusted *p*-value | Adjusted *p*-value |
| --- | --- | --- | --- | --- | --- | --- |
| ΔO_2_Hb | Walk-ST | 0.795 | 0.671 to 0.878 | 51 | <0.001 | <0.001 |
|  | Serial-7-ST | 0.790 | 0.662 to 0.874 | 51 | <0.001 | <0.001 |
|  | DTW | 0.771 | 0.630 to 0.861 | 51 | <0.001 | <0.001 |
| ΔHHb | Walk-ST | 0.781 | 0.651 to 0.869 | 51 | <0.001 | <0.001 |
|  | Serial-7-ST | 0.734 | 0.580 to 0.839 | 51 | <0.001 | <0.001 |
|  | DTW | 0.760 | 0.615 to 0.854 | 51 | <0.001 | <0.001 |

Pearson correlation coefficients (r) examining the correspondence between left and right prefrontal cortex (PFC) hemodynamic responses for each task (normal walking performed as a single task [Walk-ST], serial-7 subtraction performed as a single task [Serial-7-ST], and dual-task walking [DTW]). Correlations were calculated separately for changes in oxyhemoglobin (ΔO_2_Hb) and deoxyhemoglobin (ΔHHb). Two-sided 95% confidence intervals (CIs) for r were calculated using Fisher’s z transformation. Degrees of freedom (df) were calculated as n − 2 and were 51 for all analyses. The Hochberg procedure was applied across the six correlation tests to control the family-wise error rate. Both unadjusted and Hochberg-adjusted p-values are presented. Statistical inference was based on the adjusted p-values. The 95% CIs were not adjusted for multiple comparisons.

# 4 Supplementary Table S4. Correlations Between ΔO_2_Hb During DTW and Dual-Task Costs

| Group | Dual-task cost measure | ρ | 95% CI for ρ | Unadjusted *p*-value | Adjusted *p*-value |
| --- | --- | --- | --- | --- | --- |
| Acute stroke | Walking speed | 0.044 | −0.301 to 0.366 | 0.797 | 0.797 |
|  | Cadence | 0.049 | −0.309 to 0.387 | 0.778 | 0.797 |
|  | Stride length | −0.048 | −0.413 to 0.306 | 0.779 | 0.797 |
|  | Accuracy | −0.176 | −0.468 to 0.160 | 0.304 | 0.760 |
|  | Number of responses | −0.205 | −0.485 to 0.136 | 0.230 | 0.760 |
| Healthy older adults | Walking speed | 0.494 | 0.037 to 0.785 | 0.044 | 0.073 |
|  | Cadence | 0.542 | 0.051 to 0.790 | 0.025 | 0.073 |
|  | Stride length | 0.223 | −0.226 to 0.653 | 0.390 | 0.487 |
|  | Accuracy | 0.139 | −0.380 to 0.605 | 0.596 | 0.596 |
|  | Number of responses | −0.500 | −0.818 to 0.049 | 0.041 | 0.073 |

Spearman’s rank correlation coefficients (ρ) were calculated to examine the associations between ΔO_2_Hb during dual-task walking (DTW) and the dual-task costs of walking speed, cadence, stride length, cognitive accuracy, and number of responses. The analyses were performed separately for patients with acute stroke and healthy older adults. The 95% confidence intervals (CIs) were calculated using the bias-corrected and accelerated bootstrap method with 5,000 resamples. Because these analyses were exploratory, the Benjamini–Hochberg procedure was applied across the five correlation tests within each group to control the false discovery rate. Both unadjusted and Benjamini–Hochberg-adjusted p-values are presented. Statistical inference was based on the adjusted p-values. The 95% CIs were not adjusted for multiple comparisons. None of the associations remained statistically significant after adjustment.
